# Case Report: Pulmonary Vein Isolation as a Tailored Treatment for Recurrent Ventricular Tachycardia During Hemodialysis in a Patient With Right Coronary Artery Chronic Total Occlusion

**DOI:** 10.3389/fcvm.2022.871386

**Published:** 2022-05-30

**Authors:** Cosmin Cojocaru, Adelina Pupăză, Corneliu Iorgulescu, Sebastian Onciul, Lucian Câlmâc, Radu Vătăşescu

**Affiliations:** ^1^Department of Cardiothoracic Pathology, Carol Davila University of Medicine and Pharmacy, Bucharest, Romania; ^2^Emergency Clinic Hospital of Bucharest, Bucharest, Romania

**Keywords:** electrical storm, trigger, paroxysmal atrial fibrillation, catheter ablation, ischaemic cardiomyopathy

## Abstract

**Background:**

Catheter ablation of the ventricular substrate can reduce ventricular tachycardia (VT) recurrence and mortality in an electrical storm (ES). However, identification and specific treatment of plausible triggers is mandatory and may lead to the resolution of ES.

**Objective:**

This case presentation seeks to exemplify how pulmonary vein isolation (PVI) may represent a tailored treatment of ES in cases of ventricular substrate, which only becomes arrhythmogenic during high-rate episodes of paroxysmal atrial fibrillation (PAF).

**Results:**

A 54-year-old male with a history of inferior myocardial infarction (MI) and long-term hemodialysis was referred for repetitive implantable cardioverter-defibrillator (ICD) shocks for apparently scar-related monomorphic VT episodes preceded by PAF initiation strictly during hemodialysis. He had recently undergone ICD implantation for similar episodes of ES preceded by the rapid-ventricular response (RVR) PAF during hemodialysis. The patient had no other history of VTs. Electrocardiogram (EKG) changes occurred exclusively during PAF and suggested functional myocardial ischemia. Coronary angiography demonstrated isolated right coronary artery (RCA) chronic total occlusion (CTO). Cardiac magnetic resonance demonstrated RCA-territory residual myocardial viability and mild LV systolic dysfunction. Surgical revascularization was not feasible due to a history of bilateral above-the-knee post-traumatic amputation and severe calcification of internal mammary (IMA) and radial arteries. Subsequent CTO-percutaneous coronary intervention attempt was unsuccessful. The difficulty of assessing LV-substrate ablation end-points due to the “functional” character of the substrate, which only became arrhythmogenic during hemodialysis-related PAF, was considered. Consequently, PVI was performed rather than VT/VF substrate ablation. Twelve months after PVI, the patient remains free of PAF and VT/VF despite chronic hemodialysis sessions.

**Conclusion:**

The ES episodes can be triggered by situational factors, such as RVR-PAF and functional ischemia, during hemodialysis in patients with CTO with otherwise no episodes of VT. Tailored treatment of such factors may lead to long-term VT freedom.

## Introduction

Electrical storm (ES) can be triggered by situational factors, such as high-rate paroxysmal atrial fibrillation (PAF), in structural heart disease patients with otherwise no history of ventricular arrhythmia. Identification and tailored treatment of such triggers may lead to ES resolution (1, 2).

## Case Description

A 54-year-old male was referred for repetitive monomorphic ventricular tachycardia (VT) and ventricular fibrillation (VF) preceded by the rapid ventricular response (RVR) paroxysmal atrial fibrillation episodes (PAF), causing sudden hemodynamic instability from a hemodialysis unit. All ventricular arrhythmia episodes were preceded by AF initiation during hemodialysis (HD).

Medication included a vitamin K antagonist, ADP-inhibitor antiplatelet, beta-blocker, neprilysin inhibitor/angiotensin II receptor antagonist, central-acting alpha agonist, and a calcium blocker. Amiodarone was chronically prescribed as an antiarrhythmic drug (AAD) therapy for PAF rhythm control.

The patient had no heart failure (HF) symptoms at rest upon admission. There was a 3/6 grade aortic systolic murmur and the lung fields were clear. The patient had a long-lasting right internal jugular vein dialysis catheter and bilateral above-the-knee amputation.

The patient's physical exam did not provide evidence of acute decompensation of HF or acute coronary syndrome.

Comorbidities included PAF, hypertension, dyslipidemia, and end-stage renal disease (ESRD) undergoing HD for the past 10 years. The patient had experienced at young age extensive trauma to both inferior limbs during a mining accident, which led to rhabdomyolysis-induced renal failure.

The patient had been implanted 3 weeks prior to the current episode with a single-chamber implantable cardioverter-defibrillator (ICD) for a fast VT episode preceded by AF initiation that degenerated into VF during dialysis. Prompt resuscitation was performed. Coronary angiography documented highly calcified chronic total occlusion (CTO) of the dominant proximal right coronary artery (RCA) and insignificant asymptomatic moderate lesions in the remaining vessels.

## Diagnostic Assessment

**Figure 1:**
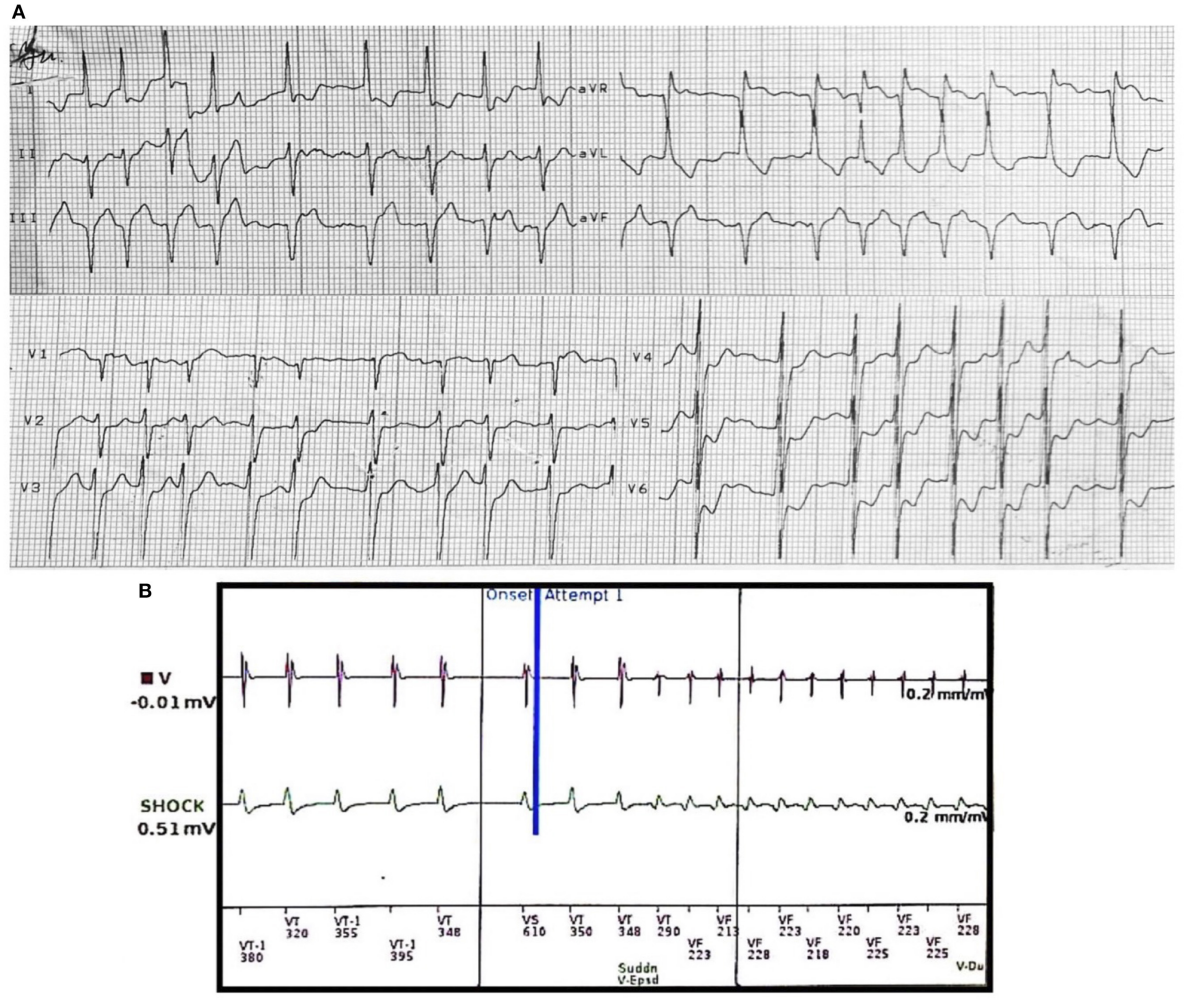
**(A)** EKG during hemodialysis prior to ventricular tachycardia initiation. Rapid ventricular response atrial fibrillation with new-onset V4–V6 ST-segment depression and aVR ST-segment elevation. **(B)** Intracardiac electrograms (EGMs) showing induction of VF-zone monomorphic ventricular tachycardia (constant cycle lengths and EGMs with new-onset morphology) after intradialytic onset of paroxysmal rapid ventricular response atrial fibrillation (irregularly irregular cycle lengths). VF, ventricular fibrillation.

The EKG performed during HD demonstrated atrial fibrillation with rapid ventricular response inferior leads Q waves, new-onset V4–V6 and D1 leads ST-depression, and aVR ST-segment elevation (Figure 1A). Sinus rhythm with frequent monomorphic premature ventricular complexes (PVCs) consistent with inferior-wall origin morphology was evident upon admission.

Device interrogation (*Inogen*™ *MINI VR, Boston Scientific*) revealed 9 similar arrhythmic events, which all responded to ICD electrical shock. Initial RVR episodes of AF (mean CL 360 ms) led to monomorphic fast VT induction (CL 220 ms), which degenerated into VF. All episodes of VT were preceded by AF (Figure 1B).

Transthoracic echocardiography (TTE) was stationary, revealing a mildly dilated and hypertrophic LV with severe inferior wall hypokinesia and mildly reduced LVEF (SBP 40–45%). LA was also dilated (93 ml, 46.5 ml/sqm). Moreover, a 70% asymptomatic stenosis of the left internal carotid artery was documented.

**Figure 2:**
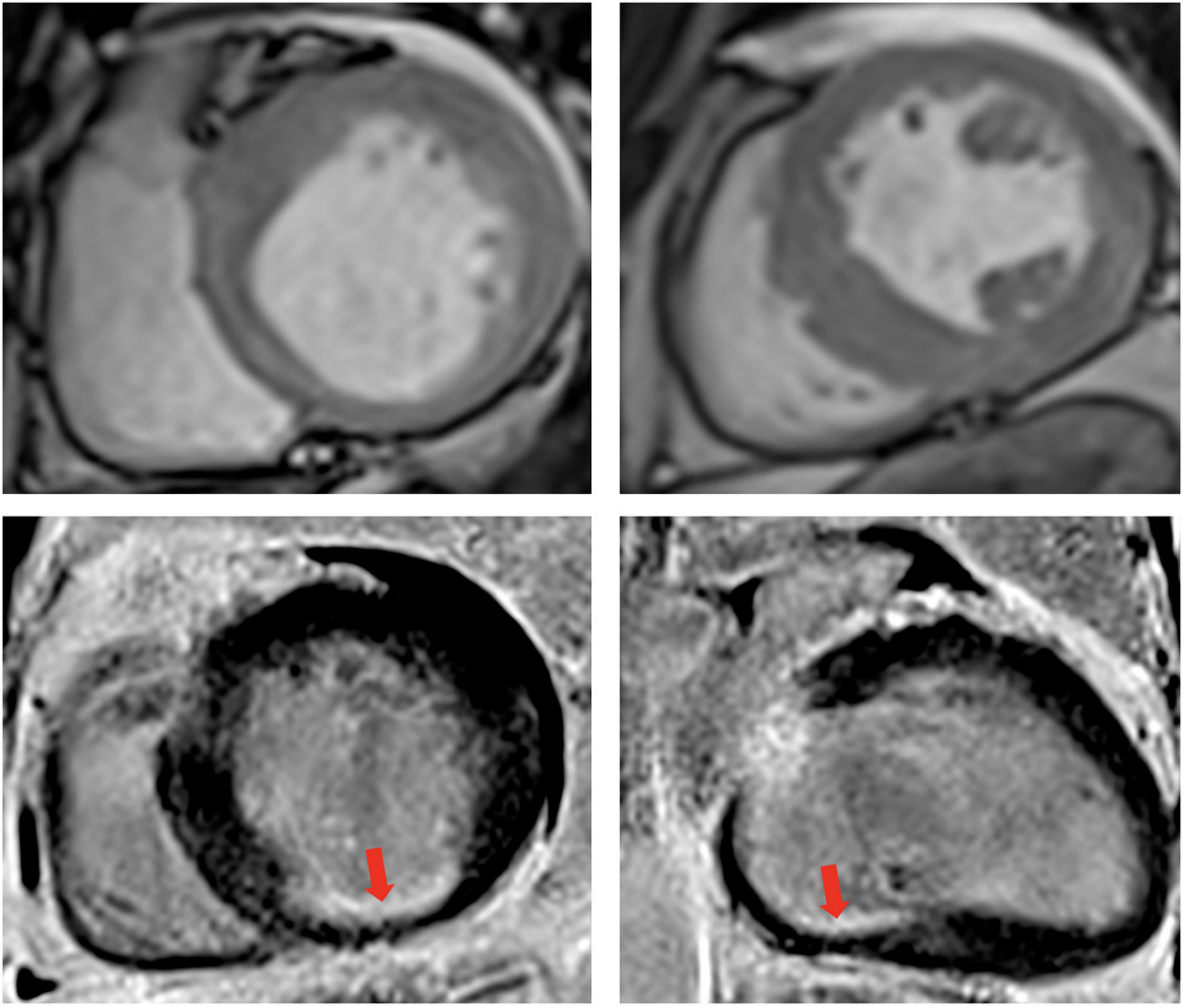
Contrast enhanced cardiovascular magnetic resonance imaging. Cine images diastolic frames in short axis at the base (top left) and at the level of papillary muscle (top right) showing asymmetrical left ventricular hypertrophy. Late Gadolinium enhancement phase sensitive inversion recovery (PSIR) imaging in short axis (Base left) and 2-chambers view (Base right), respectively, showing the subendocardial scar at the base of the inferior wall (red arrow). The transmurality of the scar was reported at 50–75%, however, note that only one segment is infarcted, while the rest of the territory supplied by the right coronary artery is viable.

Additionally, cardiac MRI (Figure 2) demonstrated LV dilation (160 ml/sqm) with mild systolic dysfunction (LVEF 41%). T1 mapping values were consistent with diffuse myocardial fibrosis (septal T1 1,100 ms). Late gadolinium enhancement (LGE) protocol revealed subendocardial scar in the basal segments of the inferior wall and the inferior septum/inferolateral wall. Minimal pericardial effusion was seen anterior and lateral to the basal LV.

Upon admission, the patient's laboratory tests revealed stationary normocytic normochromic anemia (Hb 8.9 g/dl). High-sensitivity troponin I was slightly increased (78 pg/ml). The seric potassium level was 3.92 mmol/l and the patient had no recent history of hypokalaemia. TSH, fT4, and fT3 were all in the normal range.

## Management

The patient was admitted to the intensive therapy ward. Intravenous (IV) amiodarone was initiated (bolus 10 mg/min over 30 min, maintenance rate 900 mg/24 h) in addition to metoprolol succinate (bolus 10 mg). Mild sedation was also administered (IV midazolam). Chronic medication was continued as previously mentioned.

Surgical revascularization of RCA-CTO was initially planned (euroSCORE II 3.06%). The patient was not however considered a candidate for surgical revascularization due to severe calcifications of the targetable grafts (both internal thoracic and radial arteries). Subsequently, CTO percutaneous coronary intervention (PCI) was proposed. The lesion had a J-CTO score of 4. The retrograde approach was attempted first hand and the reverse CART technique was used. However, the procedure was unsuccessful.

**Figure 3:**
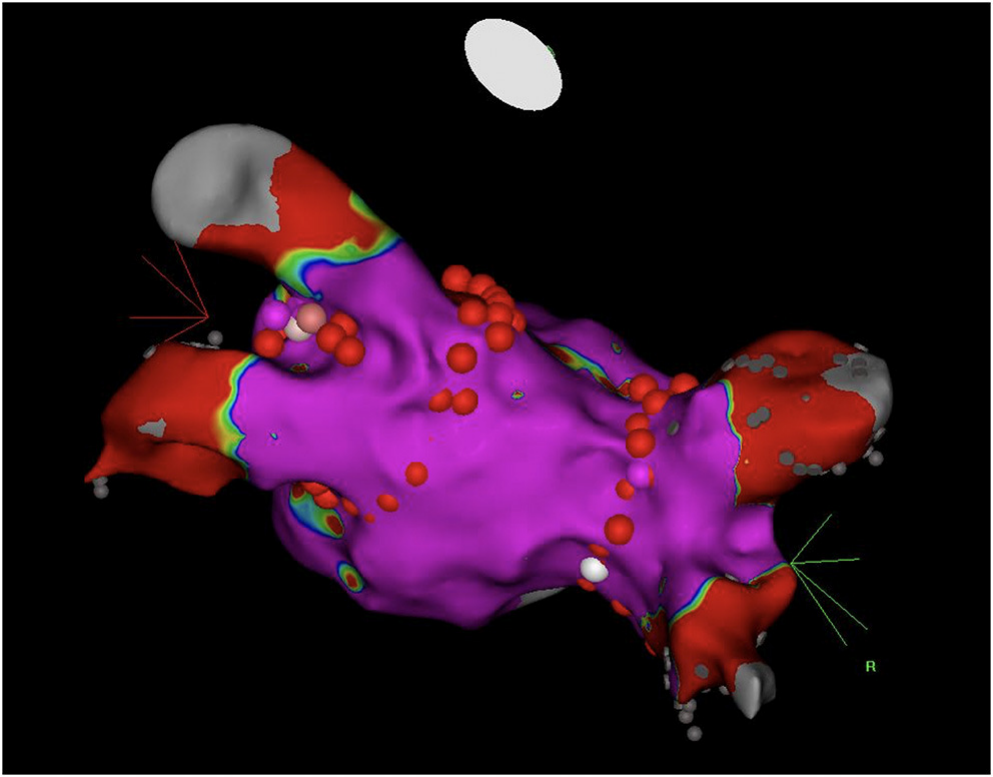
Cranial posterior aspect of the left atrium depicting the lines of antral ablation for pulmonary vein isolation. Normal myocardial voltage (>0.5 mV) is marked purple during electroanatomical reconstruction.

Consequent to RCA PCI failure, pulmonary vein isolation (PVI) by radiofrequency catheter ablation (RFCA) was preferred over performing LV substrate ablation. Wide antral circumferential ablation guided by 3-dimensional electroanatomical reconstruction was performed and a bidirectional pulmonary vein/left atrium block was obtained (Figure 3).

**Figure 4:**
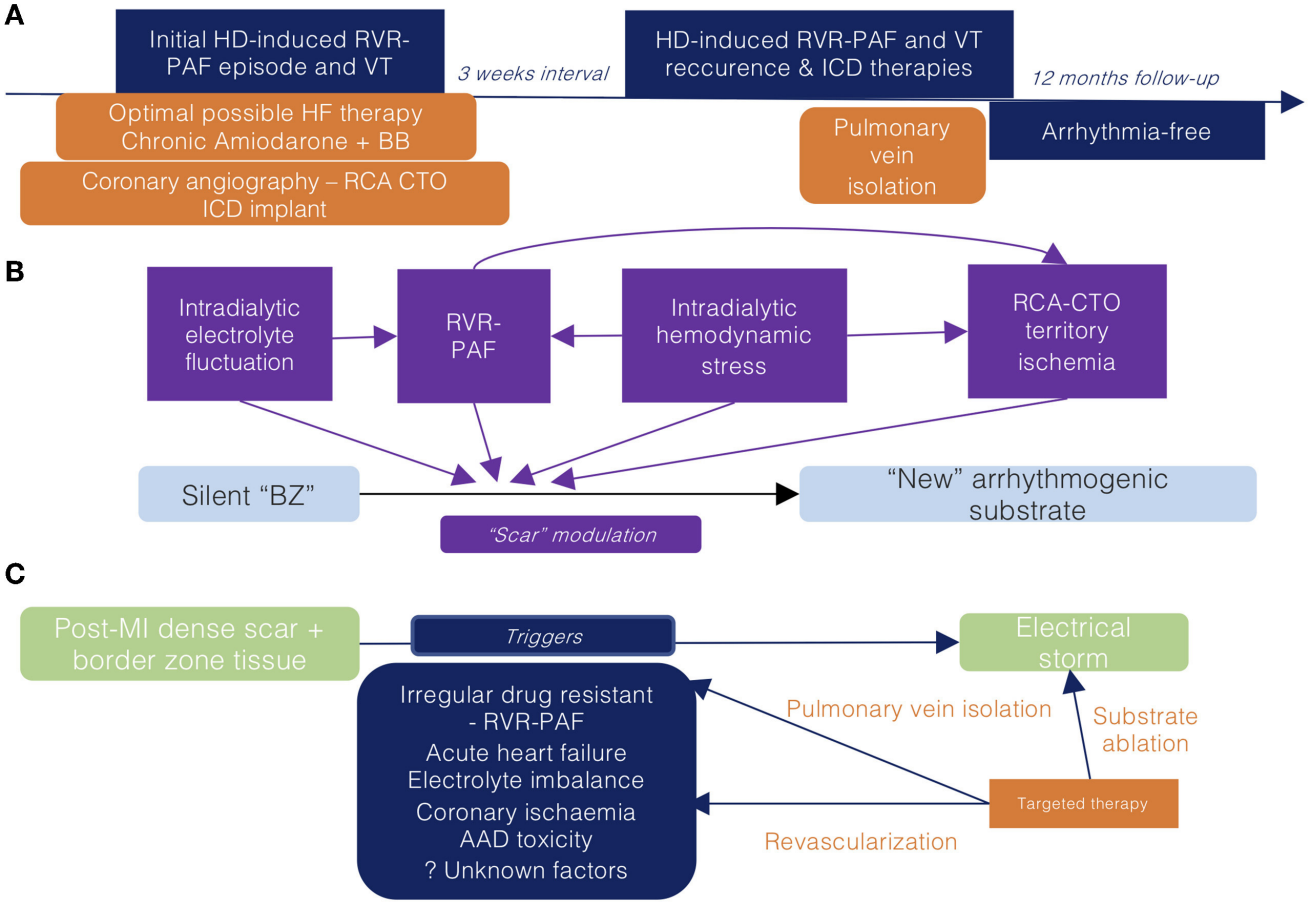
**(A)** Clinical timeline of relevant data concerning the current episode of care **(B)** Co-factors leading to scar modulation promoting ventricular arrhythmia during hemodialysis **(C)** Potential therapies targeting the multiple promoters of the current electrical storm episode. RVR-PAF, rapid ventricular response paroxysmal atrial fibrillation; HD, hemodialysis; VT, ventricular tachycardia; RCA, right coronary artery; CTO, chronic total occlusion; BZ, border zone; MI, myocardial infarction; HF, heart failure; BB, beta-blocker; AAD, antiarrhythmic drug; ICD, implantable cardioverter defibrillator.

The patient underwent HD the following day without AF or VT/VF recurrence and was discharged the following day. The patient had no recurrent episodes of AF or VT/VF throughout the first year after PVI despite programmed recurrent HD sessions (Figure 4A).

## Discussion

### Interpretation of the Occurrence of the Ventricular Arrhythmic Episodes

Multiple triggers may enable ES initiation in a previously existing structural heart disease; specific correction of such factors may improve arrhythmia management. However, their identification has been proven at best deficient in previous observational studies (3).

We considered that the arrhythmic episodes were a consequence of the progression from HD-induced hemodynamic stress to PAF, precipitating coronary hypoperfusion and leading to the further “functional” modulation of the LV post-MI scar (which was not previously clinically arrhythmogenic) (Figure 4B). Consequently, we considered that adequate control of AF episodes may provide the most benefit.

AF may be regarded as a hallmark of more advanced structural disease. This could justify the previously shown higher rate of appropriate therapies (ApT) in ICD recipients with AF episodes (4). However, AF may directly precede and “beget” VT/VF episodes (2). Furthermore, performing PVI in such patients may consequently lower the rate of ApT (1). Intradialytic AF onset is a common event in patients with ESRD (5). AF, especially, if associated with irregular RVR, may lead to impairment of coronary blood flow [even in the absence of epicardial lesions (6)] and even to increased sympathetic nerve activity (7–9). The resulting myocardial ischemia may synergically further alter conduction velocities and effective refractory periods within the scar border zone (BZ) and subsequently, promote VT induction (10).

Why the previous episodes of PAF did not induce ventricular arrhythmias is a matter of debate. We hypothesized that this may be explained by the time-dependent alteration of BZ channels (11).

Other co-factors, such as acute fluctuations of potassium and magnesium levels, may also facilitate arrhythmogenesis during HD (12, 13). Transient intradialytic hypotension may lead to RCA-CTO territory hypoperfusion and is a common event during HD (14). However, none of these had been formally documented in this particular scenario.

### Treatment Alternatives

Considering the previous interpretation of the arrhythmic substrate, we sought to evaluate treatment alternatives.

The CTO revascularization may have improved anterograde coronary blood flow during hemodynamically strenuous circumstances, such as RVR-AF or HD. However, PCI of lesions with high J-CTO scores can prove challenging and may have a lower success rate (15). Concurrently, surgical revascularization was considered impracticable due to heavy calcifications of ESRD-preferred grafts (IMA) (16).

Performing RFCA as ES therapy may improve outcomes, yet it mandates the previous correction of all potential triggers (17–19). We considered this patient exhibited a “dynamic” substrate, which alters substrate relevance in sinus rhythm. Having never experienced VTs in any other circumstances, we preferred targeting the patient's trigger for VT (AF) by PVI (Figure 3) and not the substrate itself (Figure 4C). Consequently, despite the suppression of the current AF-induced arrhythmic cluster, the persistence of inhomogeneous substrate maintains the risk of further scar-related arrhythmic recurrences.

Interestingly, left atrial voltage mapping electroanatomical reconstruction documented minimal areas of fibrosis (i.e., local EGM amplitude of <0.5 mV), which has been shown to predict favorable long-term results of PVI (20). This contrasted dilation of the left atrium and the expected diffuse fibrosis, which has most commonly been described in the ventricular myocardium in patients with ESRD (21).

In summary, if identified, tailored correction of VT-inducing mechanisms may provide long-term freedom from arrhythmia.

## Patient Perspective

Despite the firm reduction in arrhythmic death in selected high-risk populations, ICD recipients may be confronted with multifaceted psychological suffering related to their device (22–25). Our patient experienced severe anxiety and intense fear of future similar episodes after experiencing multiple internal shocks. The patient's emotional distress was most aggravated due to the potential causality relationship between hemodialysis (a mandatory recurrent medical intervention) and the arrhythmic events. This highlights the need of implementing a patient-centric comprehensive briefing program that can provide dedicated psychological support to ICD recipients.

Furthermore, successful PVI led to substantially improved self-declared quality of life both by eliminating AF burden [which has been previously shown in large-scale trials (26)] and intradialytic ICD shocks.

## Data Availability Statement

The original contributions presented in the study are included in the article/supplementary material, further inquiries can be directed to the corresponding author/s.

## Ethics Statement

The studies involving human participants were reviewed and approved by Clinical Emergency Hospital of Bucharest Ethics Committee. The patients/participants provided their written informed consent to participate in this study, for the publication of this case report, and for the publication of any potentially identifiable images or data included in this article.

## Author Contributions

CC: manuscript writing, conceptualization, and data analysis. AP: conceptualization. CI and SO: manuscript revision. LC: supervision. RV: manuscript revision, supervision, and conceptualization. All authors contributed to the article and approved the submitted version.

## Conflict of Interest

The authors declare that the research was conducted in the absence of any commercial or financial relationships that could be construed as a potential conflict of interest.

## Publisher's Note

All claims expressed in this article are solely those of the authors and do not necessarily represent those of their affiliated organizations, or those of the publisher, the editors and the reviewers. Any product that may be evaluated in this article, or claim that may be made by its manufacturer, is not guaranteed or endorsed by the publisher.
